# Environmental bacteria increase population growth of hydra at low temperature

**DOI:** 10.3389/fmicb.2023.1294771

**Published:** 2023-11-28

**Authors:** Máté Miklós, Karolina Cseri, Levente Laczkó, Gábor Kardos, Sebastian Fraune, Jácint Tökölyi

**Affiliations:** ^1^MTA-DE “Momentum” Ecology, Evolution and Developmental Biology Research Group, Department of Evolutionary Zoology, University of Debrecen, Debrecen, Hungary; ^2^HUN-REN Centre for Ecological Research, Institute of Evolution, Budapest, Hungary; ^3^National Laboratory for Health Security, Centre for Eco-Epidemiology, Budapest, Hungary; ^4^Institute of Metagenomics, University of Debrecen, Debrecen, Hungary; ^5^HUN-REN-UD Conservation Biology Research Group, Department of Botany, University of Debrecen, Debrecen, Hungary; ^6^Microbiological Reference Laboratory, National Public Health Center, Budapest, Hungary; ^7^Department of Gerontology, Faculty of Health Sciences, University of Debrecen, Debrecen, Hungary; ^8^Institute of Zoology and Organismic Interactions, Heinrich-Heine University, Düsseldorf, Germany

**Keywords:** 16S sequencing, autoclaved lake water, Cnidaria, Darwinian fitness, ecological interactions, freshwater ecology, host-associated microbiota

## Abstract

Multicellular organisms engage in complex ecological interactions with microorganisms, some of which are harmful to the host’s health and fitness (e.g., pathogens or toxin-producing environmental microbiota), while others are either beneficial or have a neutral impact (as seen in components of host-associated microbiota). Although environmental microorganisms are generally considered to have no significant impact on animal fitness, there is evidence suggesting that exposure to these microbes might be required for proper immune maturation and research in vertebrates has shown that developing in a sterile environment detrimentally impacts health later in life. However, it remains uncertain whether such beneficial effects of environmental microorganisms are present in invertebrates that lack an adaptive immune system. In the present study, we conducted an experiment with field-collected *Hydra oligactis*, a cold-adapted freshwater cnidarian. We cultured these organisms in normal and autoclaved lake water at two distinct temperatures: 8°C and 12°C. Our findings indicated that polyps maintained in sterilized lake water displayed reduced population growth that depended on temperature, such that the effect was only present on 8°C. To better understand the dynamics of microbial communities both inhabiting polyps and their surrounding environment we conducted 16S sequencing before and after treatment, analyzing samples from both the polyps and the water. As a result of culturing in autoclaved lake water, the polyps showed a slightly altered microbiota composition, with some microbial lineages showing significant reduction in abundance, while only a few displayed increased abundances. The autoclaved lake water was recolonized, likely from the surface of hydra polyps, by a complex albeit different community of bacteria, some of which (such as *Pseudomonas*, Flavobacteriaceae) might be pathogenic to hydra. The abundance of the intracellular symbiont *Polynucleobacter* was positively related to hydra population size. These findings indicate that at low temperature environmental microbiota can enhance population growth rate in hydra, suggesting that environmental microorganisms can provide benefits to animals even in the absence of an adaptive immune system.

## Introduction

Multicellular organisms are surrounded by microbes that are critically important for their health and fitness. Pathogens drive infectious diseases that weaken or kill their hosts and have been at the forefront of microbiological research in the past century (Madigan et al., 2018). A more recent discovery, host-associated microbes are involved in complex interactions with each other and their host, ultimately driving many aspects of host physiology (McFall-Ngai et al., 2013).

Pathogenic and host-associated microorganisms, just as multicellular hosts, are part of a wider network of ecological interactions with environmental microbiota, which can affect host physiology. Environmental microbes serve as a source for the assembly of host-associated microbial communities (Loudon et al., 2014; Smith et al., 2015; Knutie et al., 2017; Jani and Briggs, 2018; Prest et al., 2018; Callens et al., 2020; Singh et al., 2020; Téfit et al., 2023), which in turn have been repeatedly shown to affect host health and Darwinian fitness (Shin et al., 2011; Fraune et al., 2015; Shreiner et al., 2015; Sison-Mangus et al., 2015; Knutie et al., 2017; Gould et al., 2018; Popkes and Valenzano, 2020; Weiland-Bräuer et al., 2020). Environmental microorganisms can also directly influence host physiology by affecting immune maturation early in life, with possible fitness consequences later (Chen and Cadwell, 2022; Fallet et al., 2022; Walsh and Guillemin, 2022; Donald and Finlay, 2023; Fontaine and Kohl, 2023). Studies done on frogs, for instance, have shown that raising tadpoles in an environment with reduced microbial diversity (autoclaved lake water) disrupts their gut microbiota and affects subsequent resistance to parasitic nematode infections, likely through affecting immune maturation (Knutie et al., 2017). Likewise, raising tadpoles in water with reduced microbial diversity exhibited altered brain morphology (Emerson et al., 2023) and reduced stress tolerance under both high and low temperature stress, which ultimately impacted their fitness (Fontaine et al., 2022). All these examples resonate with the “Hygiene Hypothesis” and the recently proposed One Health principle, which posit that aspects of the wider environment are closely linked to the health of humans and animals (Yazdanbakhsh et al., 2002; Trinh et al., 2018).

While instances of environmental microbiota influencing host physiology in animals are known, the extent to which the microbial environment affects animal health and fitness remains inadequately explored. Furthermore, the range of mechanisms underlying this influence are not well-documented. Immune maturation is a recognized mechanism through which environmental microbiota can contribute to animal health, yet this mechanism is limited to vertebrates with an adaptive immune system. Invertebrates lack an adaptive immune system suggesting that immune system maturation should not depend on microbial exposure, although immune priming and immune memory might still be present in these organisms (Melillo et al., 2018; Prigot-Maurice et al., 2022). Sporadic examples of exposure to environmental microorganisms affecting animal fitness have been observed in invertebrates. For instance, in the case of the Pacific oyster *Crassirostrea gigas* microbial exposure leads to epigenetic modifications and alterations in immune gene expression, ultimately resulting in improved pathogen tolerance later in life. Yet, it is plausible that other mechanisms also play a role, some of which may not be directly related to the host’s immune system. Notably, environmental microorganisms could directly interact with host-associated microbiota in a complex ecological network (Singh et al., 2020), thereby indirectly influencing animal fitness through their impact on the host-associated microbiota.

In this study, our goal was to investigate the impact of environmental microorganisms on the fitness of animals using an invertebrate model, the freshwater cnidarian *Hydra oligactis*. Hydra polyps harbor complex microbial communities (Fraune and Bosch, 2007; Taubenheim et al., 2020) that strongly affect multiple aspects of host physiology (Fraune et al., 2015; Murillo-Rincon et al., 2017; Rathje et al., 2020), rendering them an ideal model system to understand host-microbiota interactions. To closely replicate natural conditions and understand the interactions between animals and microbes, the experiments reported here utilized field-collected specimens that hosted their natural microbial communities. These specimens were observed in controlled laboratory microcosms containing lake water sourced from their natural environment. Within this laboratory setting, hydra polyps, along with their natural microbiota, were exposed to either untreated or sterilized lake water. Hydra population size in distinct environments was used as an indicator of fitness. Further, to gain insight into the underlying dynamics of microbial communities, 16S sequencing was performed on samples collected both before and after the treatment. Based on our hypothesis, we anticipated that if exposure to environmental microorganisms has beneficial effects in hydra, the groups maintained in a natural lake water would exhibit a larger population size, while those in autoclaved lake water would display a comparatively smaller population size. Moreover, we predicted that if differences in hydra population size were influenced by host-associated microbiota, then the composition of microbial communities associated with hydra would be different depending on their respective environments (i.e., sterile or non-sterile).

## Materials and methods

### Field collection and experimental setup

All experiments reported here were performed on animals collected from a small lake in Eastern Hungary (Apagyi Kenderáztató tó; 47.9802173 N; 21.9388274E). Large numbers of *H. oligactis* can be found at this site, especially during the winter, which is the period of peak population size for this species in Hungary (M.M. & J.T., pers. obs.; see also Tökölyi, 2023 for a description of *H. oligactis* life cycle). *H. oligactis* is a psychrophilic species, i.e., it prefers colder temperatures and is able to survive and reproduce at temperatures close to freezing point, while temperatures above ~25°C are stressful to them (Bosch et al., 1988).

To understand the effect of environmental microbiota on hydra fitness, we first performed two pilot studies, the first done with animals and lake water collected on 10th January 2022, while the second performed with animals and lake water collected on 18th March 2022. Both polyps and water were collected with sterile equipment (700 mL autoclaved plastic food boxes) and brought to the laboratory in a cooled box. In the lab, lake water was first filtered through a paper filter to remove debris and zooplankton suspended in water. This non-sterile lake water served as control. Next, aliquots from this water were either autoclaved or run through a 0.22 μm PES membrane filter (FilterBio^®^, Nantong, P. R. C.) as an alternative method to remove bacteria from lake water. Finally, artificial hydra medium (Tökölyi, 2023) filtered with a 0.22 μm PES membrane filter was used as a fourth experimental group, as a control to study hydra population dynamics in an environment that is devoid of bacteria and bacterial nutrients.

From each water type, 200 mL were placed in glass containers (*N* = 5 in each group), to which we added 10 polyps. Sample size was chosen based on polyp availability. The containers were closed and placed in an incubator. To reduce stress due to temperature differences, we put polyps in incubators that had temperatures close to lake water temperature recorded during collection. Hence, in Pilot 1, animals were placed into an 8°C climate chamber, while in Pilot 2 animals were placed in a climate chamber set to 12°C. In both cases, a light/dark cycle of 16/8 h was maintained. Polyps were not fed during the experiment to avoid introducing foreign bacteria into the experimental system, but they continued to reproduce asexually after collection from energy reserves accumulated in their natural habitat. Hydra population size was recorded for a total period of 4 weeks after collection. During this period, we recorded the number of animals twice per week.

After performing and evaluating these pilot experiments, we observed a discrepancy between their results (see Results). We suspected that these discrepancies might be the result of different experimental temperatures. Therefore, in January 2023 we repeated the treatment in a main experiment, this time randomly dividing animals into 8 and 12°C treatments. We used *N* = 5 jars with 10 polyps each as starting populations size, just as in the pilot experiments. However, we did not include a sterile-filtered treatment group due to the limited availability of polyps. Recording of population size data was done for 4 weeks, twice per week.

### DNA extraction

DNA was isolated for 16S sequencing in the main experiment. First, we isolated DNA from polyps (*N* = 4) on the day of collection (24th January 2023), to describe microbiome composition of field polyps. Likewise, we isolated DNA from the 0.22 μm filters (*N* = 2) to capture microbiome composition of the water at the time of collection. Finally, from each experimental jar we extracted DNA from polyps (1 polyp/jar) and water (1 sample/jar, after filtering through a 0.22 μm PES membrane filter) at the end of the four-week observation period.

DNA was extracted through a chloroform-isoamyl alcohol extraction method (detailed description of the protocol can be found in the supplementary of Miklós et al., 2021).

### Sequencing

Concentration and purity of DNA isolates were determined using the NanoDrop One spectrophotometer (Thermo Fisher Scientific, Waltham, MA, United States). For amplification of the V1-V2 hypervariable region of the 16S rRNA gene, we used PCR primers Forward (5′- TCGTCGGCAGCGTCAGATGTGTATAAGAGACAGAGMGTTYGATYMTGGCTCAG-3′) and Reverse (5′-GTCTCGTGGGCTCGGAGATGTGTATAAGAGACAGGCTGCCTCCCGTAGGAGT-3′). We used the Phusion High-Fidelity PCR Master Mix at a final concentration of 1× with a primer concentration of 0.2 μM. For amplification of target genes, the following profile was set in a VWR Thermal Cycler UNO96 (Radnor, PA, United States): initial denaturation at 98°C for 4 min, denaturation at 98°C for 40 s, annealing at 58°C for 30 s, extension at 72°C for 25 s, and final extension at 72°C for 5 min. We checked the specificity of the amplification by running the PCR products on a 1% agarose gel and on an Agilent 2100 Bioanalyzer (Agilent Technologies, Inc., Waldbronn, Germany). We then purified the amplicons using the AMPureXP (Beckman Coulter, Brea, CA, United States) magnetic bead-based protocol. The concentration of the amplicons was normalized to 0.5 ng/μl using Qubit v4.0 (Thermo Fisher Scientific, Waltham, MA, United States) fluorometer measurements before preparation of the sequencing library. Nextera XT Index Kit v2 (Illumina, Inc., San Diego, CA, United States) was used to uniquely index each sample. Samples were then purified again using AMPure XP and quantified using Qubit v4.0 and Agilent 2,100 Bioanalyzer. The library of pooled samples was diluted to 4 nM and sequenced using MiSeq (Illumina, Inc., San Diego, CA, United States) Reagent Kit v3 with 300 bp PE option according to the manufacturer’s protocol, except for the addition of 35% PhiX standard to the library to compensate for the low complexity of the library.

### Data analyses

We evaluated the effect of water treatments on hydra population size by comparing the number of individuals/jar after 4 weeks, employing generalized linear models (GLMs) with a Poisson distribution, as implemented in R v4.3.1 (R Core Team, 2023) and pairwise *post-hoc* comparisons with Benjamini-Hochberg correction as implemented in the *emmeans* R package v1.8.7 (Lenth, 2022).

16S sequencing results underwent an initial processing step with *fastp* (Chen et al., 2018). This step involved removal of low-quality nucleotides (mean Phred score < 20 over a sliding window of 5 nucleotides) and employed the following additional parameters: qualified Phred score: 20, unqualified percent limit: 50%, base correction on overlapping reads enabled.

Subsequently, in the sequence analysis, *Qiime2* (v2023.5; Bolyen et al., 2019) was utilized to assign taxonomic information to sequences. The first task was denoising reads into Amplicon Sequence Variants (ASVs) using the *DADA2* plugin in *Qiime2* (Callahan et al., 2016). This was achieved by employing specific trimming and truncation parameters selected after reviewing positional quality plots of forward and reverse reads. The first 5 bases were trimmed from both forward and reverse reads, with forward reads truncated at 260 nucleotides and reverse reads truncated at 200 nucleotides. At this stage, samples with <5,000 remaining reads were removed.

Next, the procedure involved two major steps. Initially, the feature classifier marker genes were extracted from the Silva 138 dataset (Quast et al., 2012), using the 27F and 338R primers (27F: AGMGTTYGATYMTGGCTCAG, 338R: GCTGCCTCCCGTAGGAGT). Subsequently, a Naive Bayes classifier was trained on this region of interest of the 16S gene and taxonomy assignment of reads was performed with the resulting classifier (Bokulich et al., 2018). Finally, the resulting feature table was filtered by removing chloroplasts and mitochondria and features that did not have a relative abundance of 0.05% with a prevalence of at least 5%.

Diversity analyses were performed in R v4.3.1 (R Core Team, 2023), using the *phyloseq* v1.44 (McMurdie and Holmes, 2013), *microbiome* v1.22 (Lahti and Shetty, 2019), *qiime2R* v0.99.6 (Bisanz, 2018) and *metagMisc* v0.5.0 (Mikryukov, 2023) packages. We calculated Chao1, Shannon and Pielou evenness indices as measures of alpha diversity. As measures of beta diversity, we calculated Bray-Curtis, Jaccard presence-absence as well as Unifrac weighted and unweighted distances and used *PERMANOVA* to compare treatment groups. Differential abundance analysis was performed with *ANCOM-BC* v2.2.1 (Lin and Peddada, 2020) and *LinDA* v0.1.0 (Zhou et al., 2022) in R, two methods that were shown to reliably identify differentially regulated taxa (Nearing et al., 2022; Yang and Chen, 2023). We identified taxa as differently abundant if they had a Benjamini-Hochberg-adjusted *p-*value < 0.05 in both methods.

To identify bacterial taxa that could explain variation in population size in hydra we performed Spearman rank-correlation between population size in jars with individual microbial abundance values from either polyp or water samples. Data from both temperature groups were combined for this analysis. We performed Benjamini-Hochberg correction on the resulting *p*-values and selected taxa with an adjusted *p-*value < 0.05. Preliminary analysis uncovered one bacterial genus (*Polynucleobacter*) whose abundance on polyps predicted hydra population size, therefore we performed more in-depth analysis with this taxon. *Polynucleobacter* is a genus that has both free-living and symbiotic forms and is known to be associated with hydra (Fraune and Bosch, 2007; Fraune et al., 2010). To find out the type of *Polynucleobacter* that was found within our samples, we aligned *Polynucleobacter* sequences generated in this study to sequences obtained from hydra symbionts, ciliate symbionts and free-living forms, as well as *Raistonia basilensis* as outgroup (Fraune and Bosch, 2007; Fraune et al., 2010) with *MAFFT* (Katoh and Standley, 2013) in *Qiime2* v2023.5 and reconstructed their phylogenetic relationships with *fasttree* v2.1.11 (Price et al., 2010).

Finally, to gain insight into the functional differences caused by altered microbiota composition on polyps and in their surrounding medium, we used *Picrust2* v.2.5.2 (Douglas et al., 2020) to predict functional abundances using phylogenetic investigation of marker gene sequences. From the *Picrust2* output, *MetaCyc* (Caspi et al., 2020) pathway abundance values were extracted and further analyzed with *ANCOM-BC* and *LinDA*, as described above. We considered pathway abundances altered if they had Benjamini-Hochberg-adjusted *p*-values < 0.05 in both methods.

## Results

### Population growth of hydra in sterilized lake water

Population growth in Pilot 1 was significantly affected by water treatment (Poisson GLM, LRT, λ^2^ = 44.09, *p* < 0.001). Population size at the end of the experiment was highest in lake water and was significantly lower in hydra medium, autoclaved lake water and filtered lake water, being lowest in autoclaved lake water (Supplementary Figure S1). Water treatment also affected population size in Pilot 2 (Poisson GLM, LRT, λ^2^ = 13.86, *p* = 0.003). In this case, however, the highest population size was observed in hydra medium, while population size in autoclaved and filtered lake water was not significantly different from lake water (Supplementary Figure S2).

**Figure 1:**
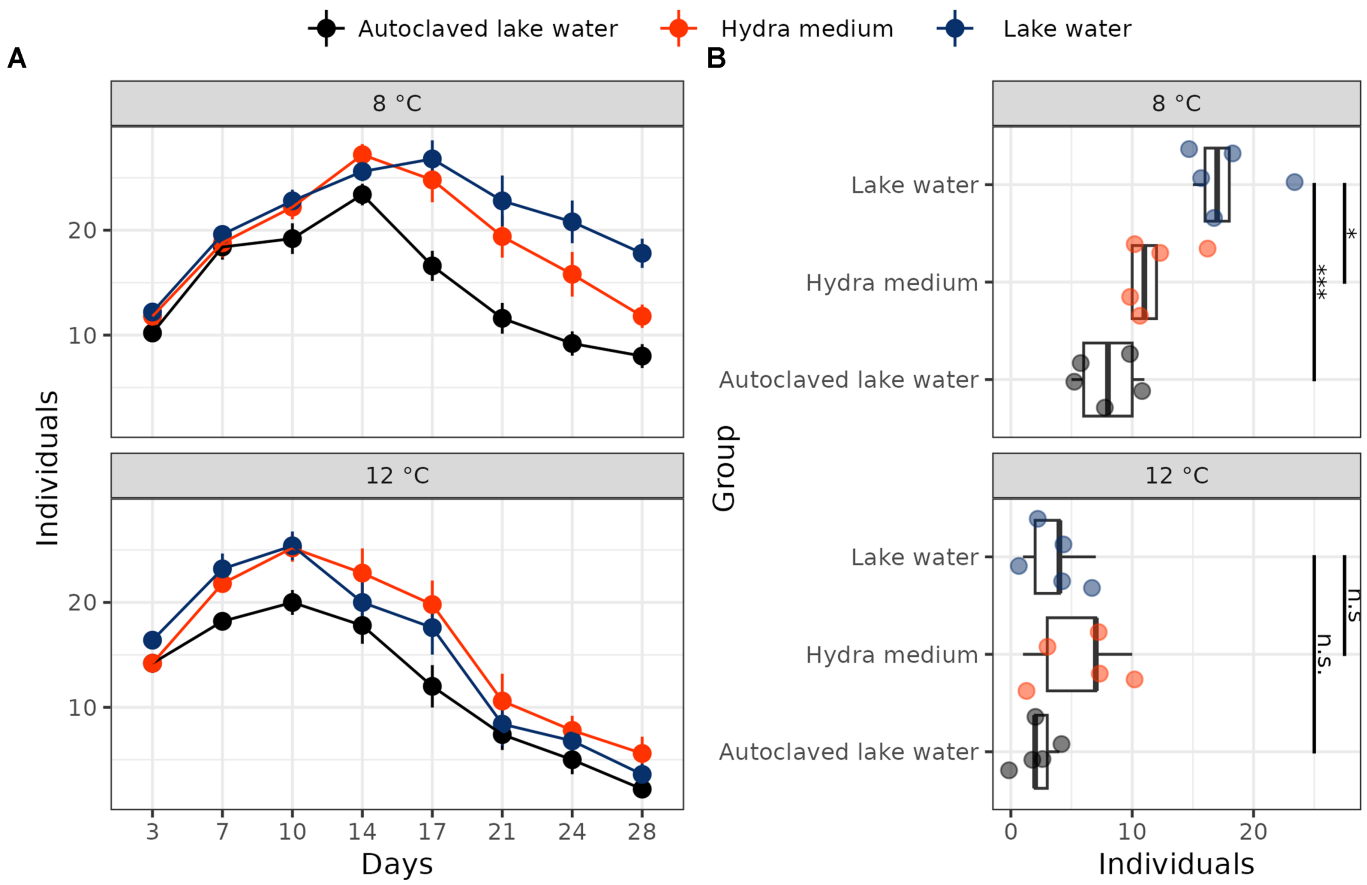
Population dynamics **(A)** and final population size **(B)** of field-collected hydra polyps cultured at 8°C and 12°C in normal lake water, autoclaved lake water or hydra medium. All treatment groups consisted of five jars with 10 polyps each as starting population size. Significance stars: ***p < 0.001; **p < 0.01; *p < 0.05; n.s. *p* ≥ 0.05. Significance was estimated with treatment vs. control *post-hoc* tests performed after Poisson GLM, with lake water as a reference level.

In the main experiment repeated with polyps collected in January 2023 and subsequently cultured at two different temperatures (8 and 12°C), we found a significant interaction between temperature and water treatment (Poisson GLM, LRT, λ^2^ = 6.35, *p* = 0.042). At 8°C, polyps in both hydra medium and autoclaved lake water had reduced population sizes compared to lake water (Poisson GLM, *post-hoc* tests with Benjamini-Hochberg correction: hydra medium: estimate = −0.41, *p* = 0.014; autoclaved lake water: estimate = 0.80, *p* < 0.001; Figure 1). By comparison, population sizes in autoclaved lake water or hydra medium did not differ compared to normal lake water at 12°C (Poisson GLM, *post-hoc* tests with Benjamini-Hochberg correction: hydra medium: estimate = −0.44, *p* = n.s.; autoclaved lake water: estimate = 0.49, *p* = n.s.; Figure 1).

### Microbiota alpha and beta-diversity on polyps and surrounding medium

**Figure 2:**
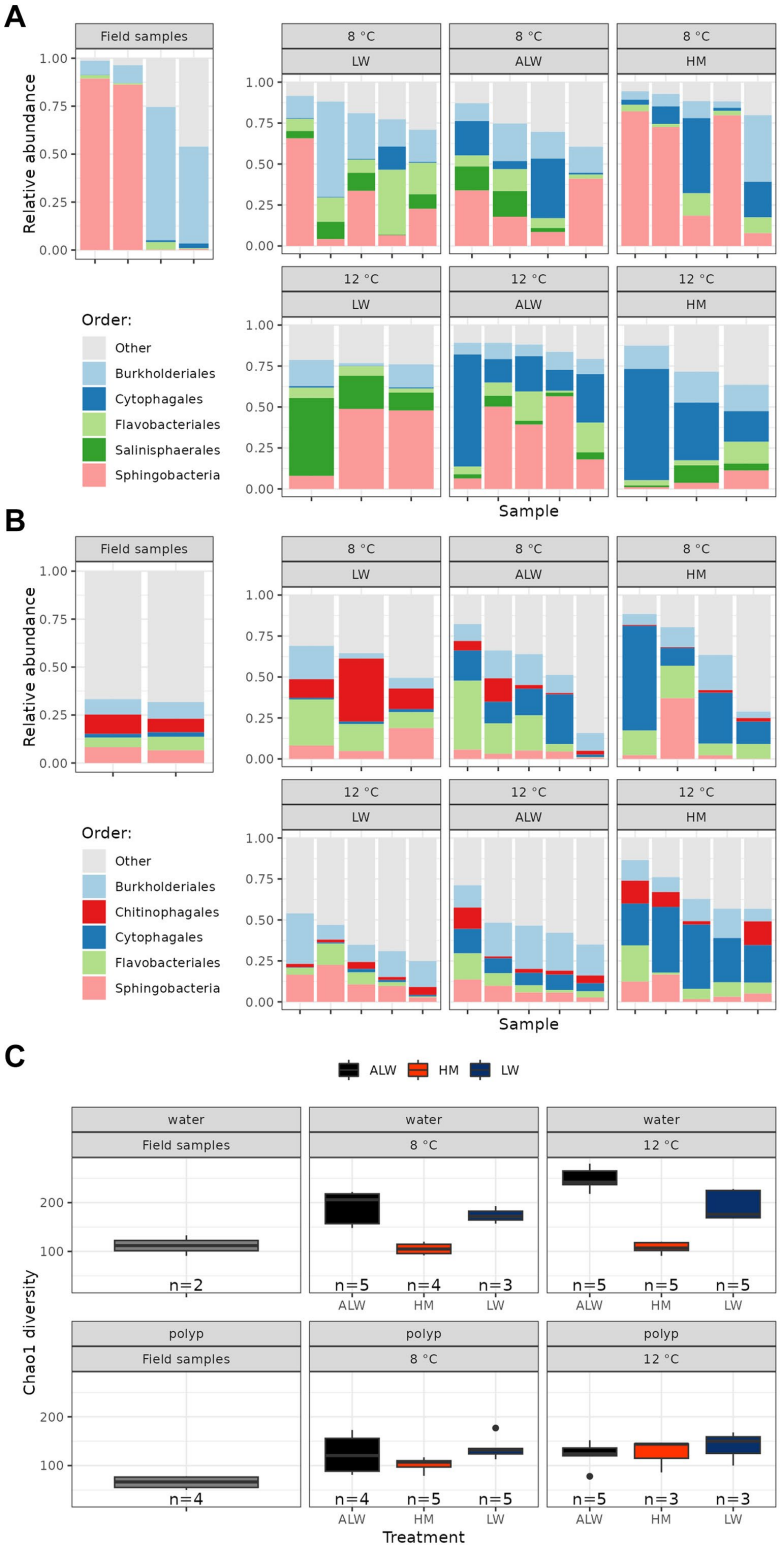
Relative abundance (percentage read counts in the whole sample) of the top five bacterial orders on polyps **(A)** and in water samples **(B)**. Alpha diversity (Chao1 index) of field samples and experimental groups is shown on panel **(C)**.

In polyps, the most abundant bacterial orders were Burkhorderiales, Cytophagales, Flavobacteriales, Salinisphaerales and Sphingobacteria (Figure 2A; the 15 most abundant genera are shown in Supplementary Figure S3). Microbiota alpha diversity (as estimated by Chao1 and Shannon indices) was highest in the field-collected individuals, while evenness did not show clear trends (Supplementary Table S1). Within experimental animals, polyps cultured in lake water had the highest, while those cultured in hydra medium had the lowest alpha diversity. Diversity was slightly larger at 12°C compared to 8°C (Figure 2C).

Water samples were overall more diverse than polyp samples (Figure 2; Supplementary Figure S3; Supplementary Table S1). The most abundant bacterial orders were Burkholderiales, Chitinophagales, Cytophagales, Flavobacteriales and Sphingobacteria (Figure 2B; the 15 most abundant genera are shown in Supplementary Figure S4). Alpha diversity was lower in field water samples compared to experimental ones (Figure 2C). Within experimental groups, diversity was lowest in hydra medium and highest in autoclaved lake water. In all three treatment groups diversity was higher at 12°C compared to 8°C (Figure 2C).

**Figure 3:**
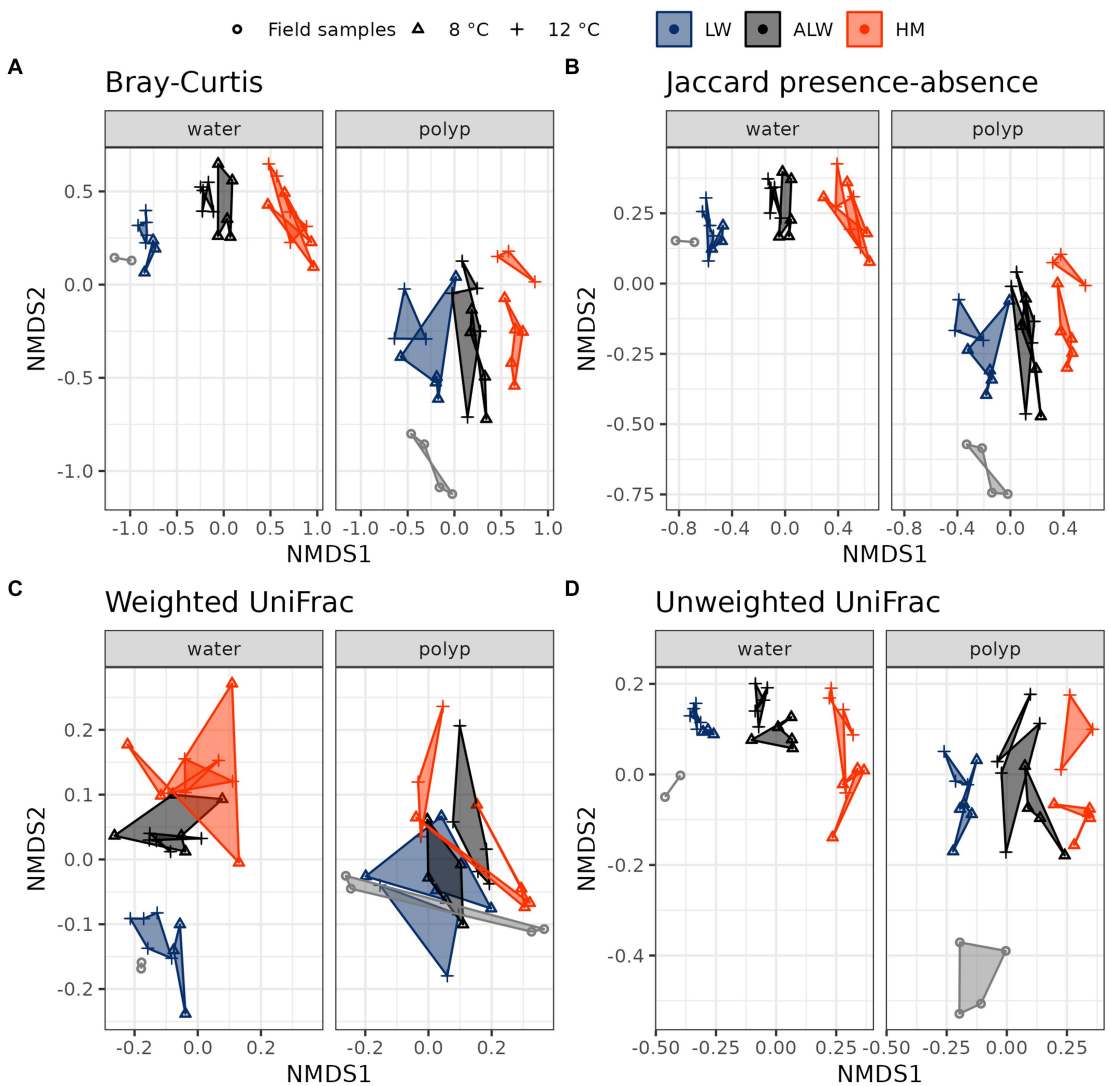
Beta-diversity differentiation of treatment groups, as measured by Bray-Curtis **(A)**, Jaccard presence-absence **(B)**, weighted UniFrac **(C)**, and unweighted UniFrac **(D)** distances.

Investigation of beta diversity showed a differentiation of water from polyp samples, field samples from experimental animals (both within polyp samples and water samples) and the three water treatment groups from each other with the Bray-Curtis, Jaccard presence-absence and unweighted UniFrac distances, while samples were broadly overlapping when comparing them with the weighted UniFrac distances (Figure 3).

Statistical comparison of treatment groups with *PERMANOVA* and pairwise Adonis showed significant differences between polyps cultured in lake water, autoclaved lake water and hydra medium in the two indices that focus on rare taxa (Jaccard presence-absence and unweighted UniFrac), a marginally significant Bray-Curtis and a nonsignificant weighted UniFrac (Table 1). Water samples showed a stronger differentiation, with all comparisons being significant, except for the weighted UniFrac distance on 8°C.

**Table 1:** Pairwise Adonis between treatment groups, using four different beta-diversity metrics.

	Bray-Curtis		Jaccard presence-absence		Weighted UniFrac		Unweighted UniFrac	
Comparison	*R* ^2^	padj	*R* ^2^	padj	*R* ^2^	padj	*R* ^2^	padj
**Polyps 8°C**								
Lake water vs. Hydra medium	0.20	0.075	**0.24**	**0.015**	0.24	0.122	**0.46**	**0.010**
Lake water vs. Autoclaved lake water	0.18	0.082	**0.21**	**0.018**	0.15	0.287	**0.40**	**0.009**
Hydra medium vs. Autoclaved lake water	0.29	0.054	**0.19**	**0.015**	0.32	0.122	**0.29**	**0.009**
**Polyps 12°C**								
Lake water vs. Hydra medium	0.32	0.100	0.32	0.100	0.35	0.100	0.44	0.100
Lake water vs. Autoclaved lake water	**0.31**	**0.050**	**0.23**	**0.024**	0.37	0.054	**0.29**	**0.023**
Hydra medium vs. Autoclaved lake water	**0.27**	**0.050**	**0.20**	**0.024**	0.33	0.069	**0.27**	**0.023**
**Water 8°C**								
Lake water vs. Hydra medium	**0.39**	**0.030**	**0.38**	**0.031**	0.38	0.056	**0.65**	**0.023**
Lake water vs. Autoclaved lake water	**0.41**	**0.024**	**0.34**	**0.027**	0.37	0.056	**0.46**	**0.023**
Hydra medium vs. Autoclaved lake water	**0.24**	**0.018**	**0.25**	**0.021**	0.16	0.241	**0.40**	**0.012**
**Water 12°C**								
Lake water vs. Hydra medium	**0.38**	**0.012**	**0.32**	**0.011**	**0.55**	**0.012**	**0.62**	**0.010**
Lake water vs. Autoclaved lake water	**0.47**	**0.016**	**0.34**	**0.011**	**0.52**	**0.012**	**0.53**	**0.010**
Hydra medium vs. Autoclaved lake water	**0.30**	**0.012**	**0.29**	**0.011**	**0.39**	**0.027**	**0.45**	**0.010**

Significant differences are highlighted in bold.

### Differentially abundant taxa and functional differences

To identify bacterial taxa that might explain the lower population size of hydra in sterilized lake water, we performed differential abundance analysis, comparing microbiota composition (read counts) of lake water vs. autoclaved lake water with *ANCOM-BC* and *LinDA*.

**Figure 4:**
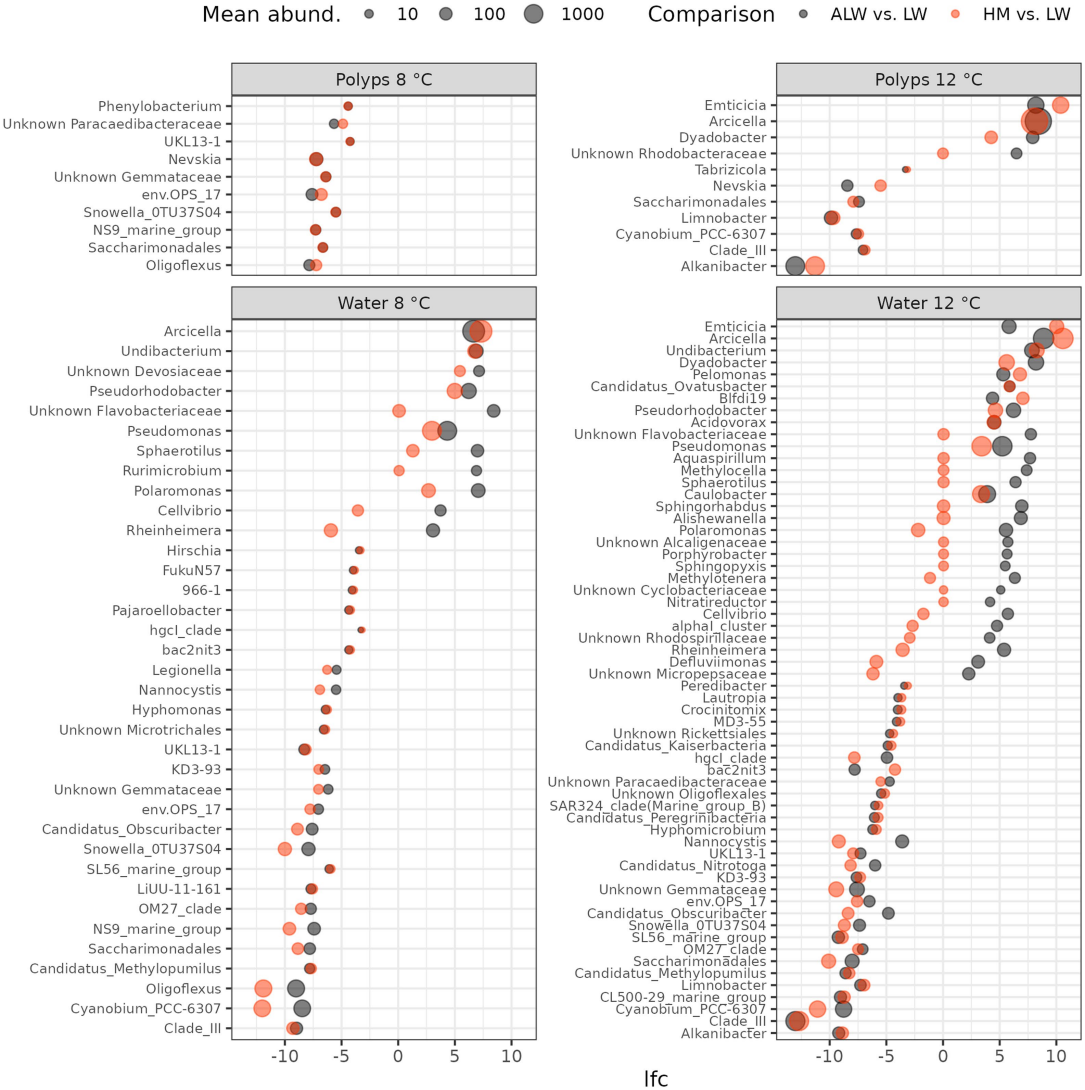
Log2-fold change (lfc) of bacterial taxa identified to be differentially abundant by both *ANCOM-BC* and *LinDA*. Black dots show lfc of bacterial abundance from lake water to autoclaved lake water, while red dots show the change from lake water to hydra medium. Symbol size is proportional to the mean abundance of bacterial genera across samples.

Polyps showed fewer differentially abundant features than water samples: only 10 and 11 taxa were identified to be differently abundant between autoclaved lake water and normal lake water at 8 and 12°C, respectively (Figure 4). Most of these were reduced read counts of bacteria in autoclaved lake water (with the exception of *Emtiticia*, *Arcicella*, *Dyadobacter* and an unknown Rhodobacteraceae, which had higher read counts in autoclaved lake water). However, even in most of these taxa, the same pattern was observed in the comparison between hydra medium and lake water (i.e., higher read counts in hydra medium than lake water), suggesting that they were not specifically associated with sterile lake water.

In contrast to polyps, water samples showed a much higher number of differentially abundant taxa: 36 and 60, respectively in the 8 and 12°C groups, identified by both *ANCOM-BC* and *LinDA*. Overall, a slightly larger proportion of taxa were less abundant in autoclaved lake water (25/36 at 8°C and 30/60 at 12°C). Top taxa (based on *LinDA* log2-Fold Change values) with significantly higher read counts in autoclaved lake water were: *Arcicella*, *Undibacterium*, unknown Devosiaceae on 8°C and *Emtiticia*, *Arcicella* and *Undibacterium* at 12°C. Many of the taxa identified to be differently abundant in autoclaved lake water showed the same difference from lake water in hydra medium. However, some of them on both temperatures were differentially abundant in autoclaved lake water, while showing a lower log2-Fold Change value in hydra medium (unknown Flavobacteriacieae, *Pseudomonas*, *Pseudorhodobacter*, *Sphaerotilus*, *Rurimicrobium*, *Polaromonas*, *Cellvibrio*, *Rheinheimera*; Figure 4).

**Figure 5:**
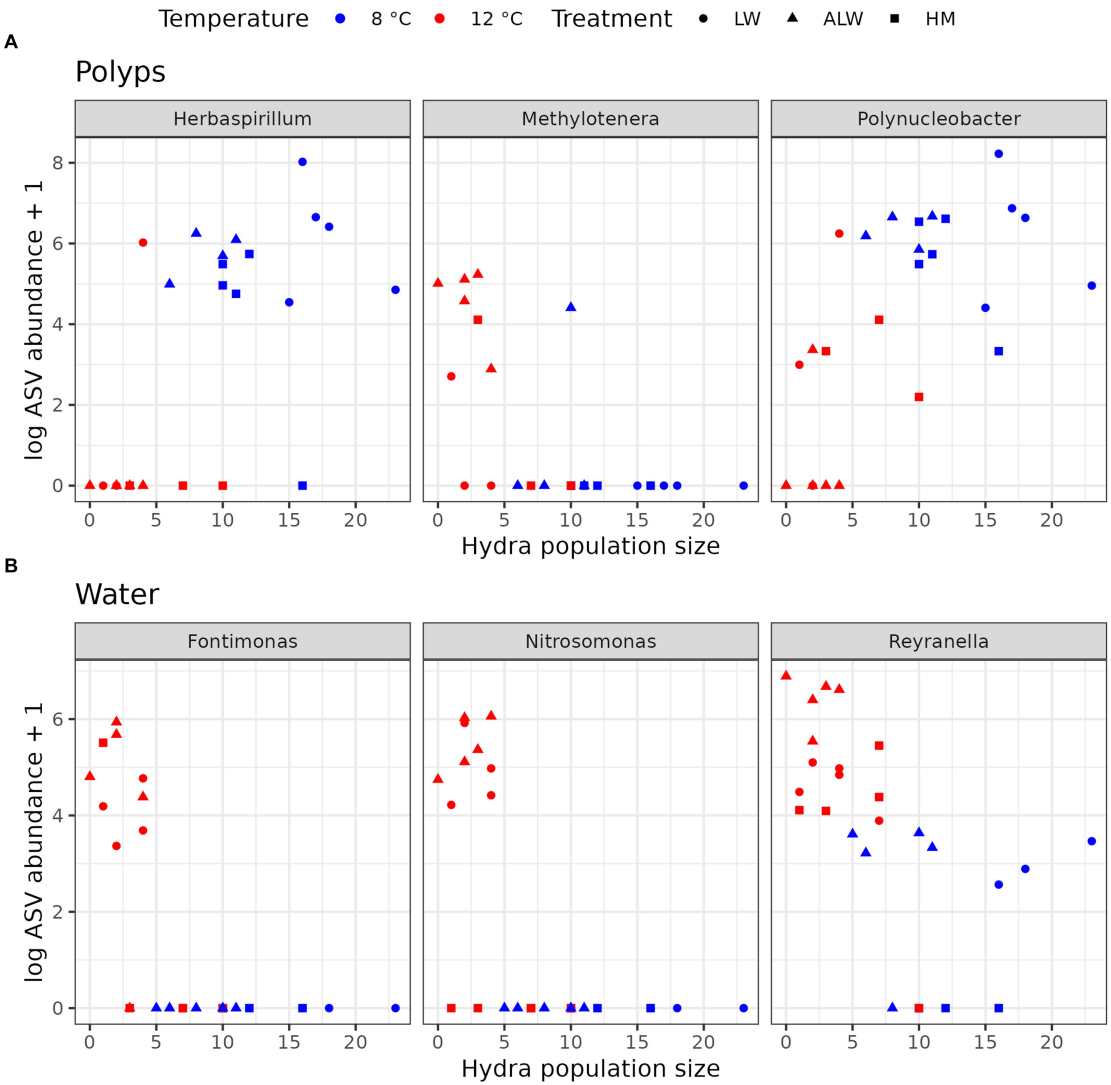
Bacterial genera from polyps **(A)** and water **(B)** whose abundance significantly correlated with hydra population size. Correlations were performed with Spearman’s method, followed by p-adjustment with Benjamini-Hochberg’s method.

**Figure 6:**
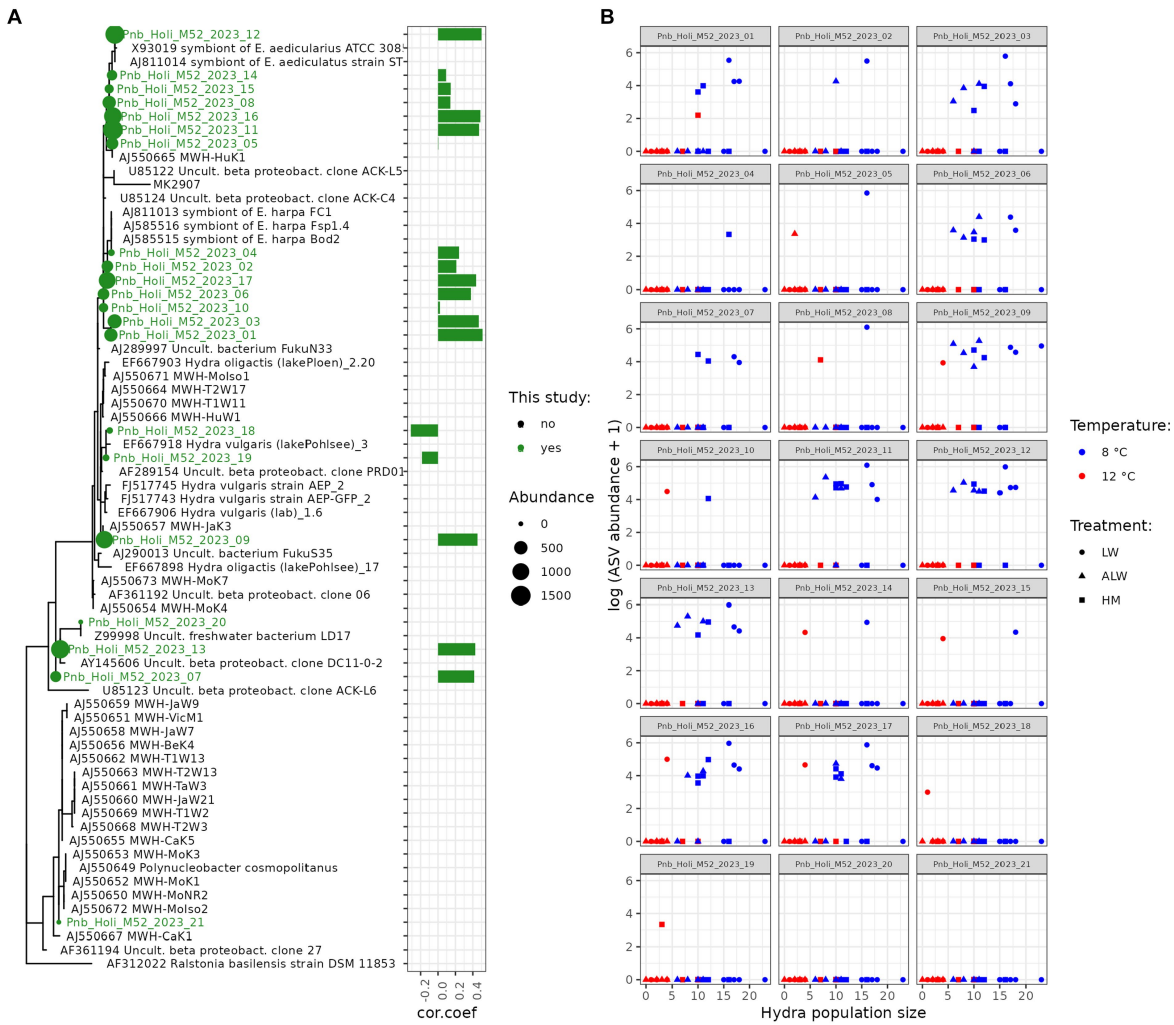
Phylogenetic relationship **(A)** of *Polynucleobacter* ASVs detected in this study (highlighted in green) to other *Polynucleobacter* taxa. The full tree contains in addition bacteria that are known symbionts of the ciliates *Euplotes aedicularius* or *E. harpa*, symbionts of *Hydra vulgaris* strain AEP, various hydra associated *Polynucleobacter* sequences, as well as free-living forms. Bars show the Spearman-rank correlation coefficient between population size and ASV abundance. Panel **(B)** shows scatterplots of individual *Polynucleobacter* ASVs on polyps with hydra population size in the experimental units.

To further gain insight into predictors of hydra population size across different treatments, we correlated population size on Day 28 from all treatments combined with read counts aggregated at the genus level. This analysis selected three bacterial taxa found on polyps that correlated with hydra’s final population size: *Polynucleobacter* and *Herbaspirillum* positively and *Methylotenera* negatively (Figure 5A). In the case of water microbiota, three taxa were found to be significantly correlated with hydra population size (all three negatively): *Reyranella*, *Nitrosomonas*, and *Fontimonas* (Figure 5B). Since *Polynucleobacter* was previously found to be associated with hydra (Fraune and Bosch, 2007; Fraune et al., 2010), we performed phylogenetic reconstruction on the ASVs sequenced from our samples. There was a total of 21 individual *Polynucleobacter* ASVs in our samples, which on polyps varied in read counts from 0 to 1,514 (mean abundance 511.5). Most of these ASVs clustered tightly with sequences isolated from ciliate and hydra samples, although they did not form a monophyletic lineage (Figure 6A). Correlating individual *Polynucleobacter* ASVs with hydra population size revealed that no single ASV determined the positive relationship between *Polynucleobacter* and hydra population size, but rather multiple ASVs contributed to that pattern (Figure 6B).

**Figure 7:**
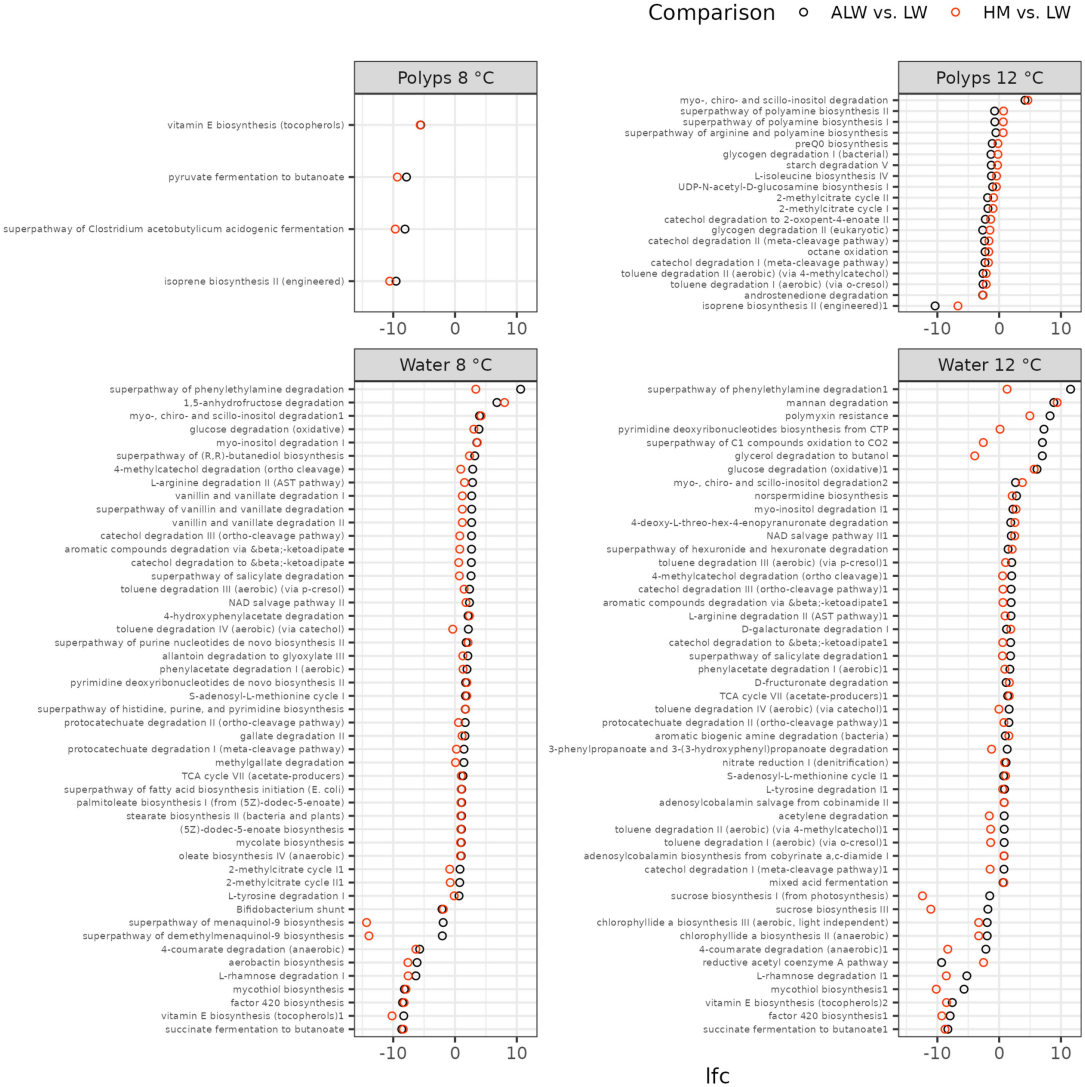
Functional changes associated with altered microbiota composition, as identified by *Picrust2*. The graph shows log2-fold change (lfc) of MetaCyc pathway abundances, compared with *ANCOM-BC* and *LinDA*. Only pathways that emerged significant in both methods are shown. Black dots show lfc of bacterial abundance from lake water to autoclaved lake water, while red dots show the change from lake water to hydra medium.

Finally, we analyzed patterns of functional abundance in polyps and their surrounding medium with *Picrust2*. Only a few *MetaCyc* pathways were significantly altered in polyps, and – with one exception – all of these were down-regulations (Figure 7). By comparison, a large number of pathways were up regulated in autoclaved lake water compared to normal lake water on both temperatures. Many of these pathways are involved in degradation of organic molecules (e.g., phenylethylamine, inositol, glucose, mannan, glycerol, etc.). Furthermore, many of them were specifically up-regulated only in autoclaved lake water but not hydra medium (Figure 7). In addition, several biosynthesis pathways (e.g., vitamin E, mycothiol) were starkly downregulated in autoclaved lake water on both temperatures.

## Discussion

### Population growth of hydra is positively affected by environmental bacteria

Environmental microbiota is considered important for animal health because of its contribution to proper immune maturation in animals possessing an adaptive immune system. While research in vertebrates has unveiled numerous instances where the presence of environmental microbes can positively affect animal health (Knutie et al., 2017; Fontaine et al., 2022; Emerson et al., 2023), less is understood about their role and potential fitness consequences in invertebrates that lack an adaptive immune system.

In this study, we utilized the freshwater cnidarian *Hydra oligactis* to demonstrate the significance of environmental microbiota in determining animal fitness and population dynamics. Culturing polyps in lake water sterilized through autoclaving resulted in reduced population growth compared to normal lake water at 8°C, although not at 12°C. The decreased population growth might be attributed to a lower asexual budding rate or an increased polyp mortality rate (or both) in the sterilized environment. Although we are currently unable to differentiate between these possibilities, we did observe a substantial number of diseased and disintegrating polyps in the autoclaved lake water treatments, indicating a likely increase in mortality.

Importantly, the same pattern of reduced population size in autoclaved lake water was observed in two independently replicated experiments conducted 1 year apart, making it unlikely that chance events were responsible for these trends. One could argue that autoclaving itself could have generated an artificial effect. Autoclaving not only kills living organisms in the water but also alters its chemical composition through precipitation of salts, some of which might be essential for hydra. However, our pilot study revealed reduced population growth in lake water that was treated differently to remove bacteria (filtered through a 0.22 μm membrane filter), which refutes the possibility of an autoclave-specific effect. Instead, our data implies that the presence of environmental microbiota is advantageous for hydra.

There are several, mutually non-exclusive, explanations for a positive effect of environmental microbes on hydra. First, competitive interactions might exist between environmental and host-associated microorganisms, with downstream consequences on the host. For instance, a healthy environment with a diverse microbial community could harbor taxa that utilize nutrients from the water, thereby limiting population growth of potentially harmful host-associated bacteria that utilize nutrients in the water as an alternative or supplementary growth source. Upon the removal of these environmental microbes, bacteria on the host can multiply and outcompete beneficial microbes, resulting in dysbiosis, and ultimately, death of polyps (Lachnit et al., 2019).

An alternative competitive scenario arises when environmental microbiota, through occupying the space between polyps (i.e., the water), restrain the spread of pathogens. If a bacterium can utilize both animal tissue and complex macromolecules in the water for growth, then the reduced competition from environmental microbiota in autoclaved lake water could expedite the spread of this bacterium from one host to another. The existence of these competitive interactions between environmental and host-associated microbes is currently not well understood and further studies are required to understand ecological interactions between microorganisms associated with the host and those residing in the surrounding environment.

Finally, environmental microbiota might provide health benefits from an entirely different, non-competitive perspective, for example, by degrading toxins in the environment, such as those produced by cyanobacteria (Kato et al., 2007).

### Environmental microbiota influence host-associated bacterial diversity

To investigate which (if any) of these mechanisms might be involved and to gain insight into the potential competition between environmental and host-associated bacteria, we compared microbiota communities on polyps and their surrounding medium, both from field-collected samples and experimental ones. In examining the 8°C samples, where the sterile environment effect was observed, we noted that microbiota communities of polyps cultured in autoclaved lake water showed only slight changes compared to those of lake water-cultured animals. Although there were no substantial differences in alpha diversity, beta diversity differed when using presence-absence indices (Jaccard and unweighted UniFrac) and showed marginally significant differences when using the Bray-Curtis index. These findings suggest alterations in the composition of the microbiota community, particularly concerning rare taxa. However, the differential abundance analysis revealed only a few taxa with significant shifts in abundance among treatment groups. This finding argues against a major dysbiosis of polyps, although we cannot dismiss the possibility that some rare taxa may have had significant phenotypic effects on hydra. Additionally, we must consider that the most severely affected polyps were likely dead at the time of sampling, potentially eliminating individuals with the most dysbiotic microbiota composition.

A more pronounced restructuring of microbial communities was observed in the surrounding medium. Similar to the polyps, alpha diversity in the water at the end of the experiment was considerably higher in the experimental groups compared to the field samples which reflect the starting condition. This observation might be unexpected, considering that water samples for the experiment were collected from the same source where initial diversity estimates were made. We suspect the higher alpha diversity of experimental samples compared to field ones might be explained by the temperature differences between field and laboratory conditions. The water temperature at the time of collection was around 3–4°C, from which we transferred water to 8 and 12°C, where it was maintained for 4 weeks. This temperature increase may have facilitated the expansion of some rare taxa that were below detection level in the field samples. Moreover, alpha diversity tended to be higher at 12°C, further supporting the influence of temperature. It is known that temperature shifts (both warming and cooling) can affect microbiota composition (Li et al., 2023), yet our understanding of microbiota variation in Hungarian lakes is limited, preventing us from validating the assumption that temperature changes caused the observed patterns in microbial alpha diversity.

Interestingly, the alpha diversity in autoclaved lake water was as high as that in normal lake water, suggesting that a complex community of microbes had recolonized the empty niches of the sterilized lake water. Since everything was sterile, the only possible source of recolonization could have been from the surface of polyps. However, autoclaving might have preserved some of the DNA in lake water and we cannot assert with complete certainty that all the 16S sequences observed there originated from live bacteria, although DNA isolation was done 4 weeks after autoclaving and most DNA macromolecules should have degraded by then. Furthermore, we confirmed the presence of living microbes in significant numbers in autoclaved lake water by spreading autoclaved lake water samples on R2a agar plates (results not shown).

### Decreased population growth in autoclaved water is correlated to both the increase and decrease of specific bacterial taxa in the medium

The microbial communities in autoclaved lake water, however, differed from all other groups. The differential abundance analysis unveiled several taxa that exhibited significantly reduced abundance in autoclaved lake water. Most of these taxa also demonstrated reduced abundance in hydra medium, suggesting that they could not recolonize the competition-free environment, potentially because they were not found on the surface of polyps. Nonetheless, certain bacterial taxa exhibited increased abundance in autoclaved lake water compared to normal lake water. Many of these taxa were similarly abundant in hydra medium, implying that their rise in abundance resulted from the absence of competition from environmental microbes. Notably, members of Spirosomaceae (e.g., *Emcicia*, *Arcicella*, *Dyadobacter*) were prominent among these bacteria, displaying high abundance and large log2-Fold Changes in comparison to normal lake water. However, since these taxa changed concurrently in both autoclaved lake water and hydra medium, it is improbable that they were the cause of the reduced population growth rate of hydra in autoclaved lake water.

Conversely, several taxa experienced a more pronounced increase in abundance in autoclaved lake water than in hydra medium (an unknown Flavobacteriaceae, *Pseudomonas*, *Pseudorhodobacter*, *Sphaerotilus*, *Rurimicrobium*, *Polaromonas*, *Cellvibrio*, *Rheinheimera*). These could be bacteria that feed on organic material accumulated from dead cells in lake water and some of them might be detrimental, or even pathogenic to hydra. *Pseudomonas* and *Flavobacterium*, in particular, are known to contain opportunistically pathogenic forms in freshwater (Bernardet and Bowman, 2006; Austin, 2011). Supporting the notion of a potential detrimental effect of bacterial communities developing in a competition-free nutrient-rich environment (autoclaved lake water) a large number of degrading functional pathways were overrepresented in autoclaved lake water. However, it is currently unclear whether these degrading pathways are in any way detrimental to hydra.

By contrast, the accumulation of specific taxa in the competition-free environment is not the sole mechanism with potentially adverse effects on hydra. Such effects could be also instigated by the removal of beneficial microbes. Specifically, we found that biosynthesis of vitamin E was downregulated in autoclaved lake water at both temperatures (and even in polyps cultured on 8°C). This reduction in vitamin E could potentially be the reason for the increased mortality of polyps in autoclaved lake water, a hypothesis that could be explored in the future through the addition of vitamin E in autoclaved lake water.

### Increased temperature overrides the positive effects of environmental bacteria

Surprisingly, we did not observe a sterile environment effect when the polyps were cultured on 12°C. Remarkably, this was not because polyps in sterile water had a higher population size, but because polyps cultured in lake water exhibited a reduced population size compared to those cultured on 8°C. Such a large effect resulting from a mere 4°C change in temperature might seem unexpected. When comparing the microbiota composition of autoclaved lake water with that of normal lake water at 12°C, we did not identify marked differences between the two temperatures, although distinctions among groups were more pronounced at the higher temperature in contrast to 8°C: differentiation in beta diversity was greater and the number of differentially abundant taxa was also higher both on the polyps and in the water. This observation might be attributed to the temperature effect discussed above, i.e., microbial proliferation could be higher at increased temperature, thereby amplifying differentiation of microbial communities. However, no clear pattern emerged that could explain the temperature disparities in population dynamics. Nevertheless, it is important to emphasize that the polyps studied here were maintained without food, indicating they were under nutrient stress, and the observed patterns need to be interpreted with this information in mind. A 4°C increase in temperature under nutrient stress is likely to substantially elevate the metabolism (especially catabolic processes) in hydra and these heightened metabolic costs could potentially mediate the differential population response at different temperatures.

### Host-associated *Polynucleobacter* abundance correlates with higher population size

In an effort to consolidate the findings obtained at different temperatures and identify potential bacterial drivers of hydra population size, we correlated bacterial abundances from both polyps and water with hydra population size in the experimental units. Only a few taxa emerged to be significantly associated with hydra population size. Most of these were negative predictors and most of these occurred only in a subset of samples, suggesting that they might be spurious correlations. However, one particular taxon, *Polynucleobacter,* emerged as significantly and positively linked to hydra population size across all treatment groups, as measured in polyps.

*Polynucleobacter* is a bacterium that exists both as free-living forms and in symbiosis with ciliates, where it benefits the host fitness by correcting a genetic defect in gluconeogenesis (Vannini et al., 2007). Symbiotic forms of *Polynucleobacter* cannot be cultivated on artificial media while non-symbiotic forms are easily culturable (Vannini et al., 2007). It is also recognized as a common, intracellular symbiont of hydra, being vertically transmitted through eggs (Fraune et al., 2010), suggesting its mutualist relationship with hydra. Within our polyp samples it ranked as the 15^th^ most abundant taxon, with a reduced abundance at 12°C. Intriguingly, multiple forms of *Polynucleobacter* were discovered, some of which clustered together with sequences isolated from ciliates, and others to bacteria isolated from hydra, suggesting the presence of multiple alternative forms within hydra. The polyphyletic nature of *Polynucleobacter* ASVs identified in our samples could indicate symbiont switching between Hydra and ciliates, although we cannot dismiss the presence of ciliates (and their symbionts) in our samples, as hydra are known to harbor commensal ciliates that accumulate on them in large numbers under specific conditions (Boutry et al., 2022). If *Polynucleobacter* indeed benefits the hydra’s fitness, variation in its abundance might account for the differences in population size observed in this experiment, or at least contribute to the temperature effects observed.

## Conclusion

In summary, our findings demonstrate that culturing hydra polyps in a sterilized environment adversely affects their fitness and reduces the population growth rate at low, although not higher temperature. We propose four distinct, albeit mutually non-exclusive, mechanistic hypotheses to account for this pattern: (1) dysbiosis of polyps and their microbial communities in a competition-free, nutrient-rich environment; (2) increased proliferation of pathogenic bacteria; (3) depletion of bacteria that produce essential nutrients (e.g., vitamin E) for hydra from the external environment and (4) reduction of beneficial host-associated microbes due to altered competitive interactions with environmental microbiota. Based on the 16S data, dysbiosis of host-associated microbiota seems improbable, as we observed only minor differences in microbiota composition of polyps in sterilized vs. normal lake water. An accumulation of degrading bacteria (such as *Pseudomonas* or unknown Flavobacteriaceae) in a sterilized environment could explain the observed pattern. Additionally, we observed a decline of vitamin E biosynthesis pathways on both polyps and their surrounding environment in the sterile environments. We identified one taxon (*Polynucleobacter*), that exhibited a clear positive correlation with hydra population size. Further investigations will be necessary to thoroughly examine these mechanistic explanations.

## Data availability statement

Sequence data generated for this study can be found in the SRA archive under BioProject accession number PRJNA1007933 (https://www.ncbi.nlm.nih.gov/bioproject/PRJNA1007933). Population size data and code used for all analyses is available at: https://github.com/jtokolyi/Hydra-SterileEnv.

## Ethics statement

Ethical approval was not required for the study involving animals in accordance with the local legislation and institutional requirements because experiments were performed with a non-protected invertebrate (*Hydra oligactis*).

## Author contributions

MM: Conceptualization, Data curation, Investigation, Methodology, Project administration, Resources, Writing – original draft, Writing – review & editing. KC: Investigation, Methodology, Writing – original draft, Writing – review & editing. LL: Formal analysis, Investigation, Methodology, Project administration, Writing – original draft, Writing – review & editing. GK: Funding acquisition, Investigation, Methodology, Project administration, Resources, Writing – original draft, Writing – review & editing. SF: Conceptualization, Formal analysis, Methodology, Visualization, Writing – original draft, Writing – review & editing, Funding acquisition. JT: Conceptualization, Data curation, Formal analysis, Funding acquisition, Investigation, Methodology, Project administration, Resources, Software, Supervision, Validation, Visualization, Writing – original draft, Writing – review & editing.
