# Supplementary material for: Environmental bacteria increase population growth of hydra at low temperature
Source: Front Microbiol. 2023 Nov 28;14:1294771. doi: 10.3389/fmicb.2023.1294771 (PMC10715282; doi:10.3389/fmicb.2023.1294771)
Supplement: Supplementary file 1 [file Data_Sheet_1.docx]

Supplementary Material

# Supplementary Data

# Supplementary Figures and Tables

## Supplementary Figures

**2.2 Supplementary Tables**


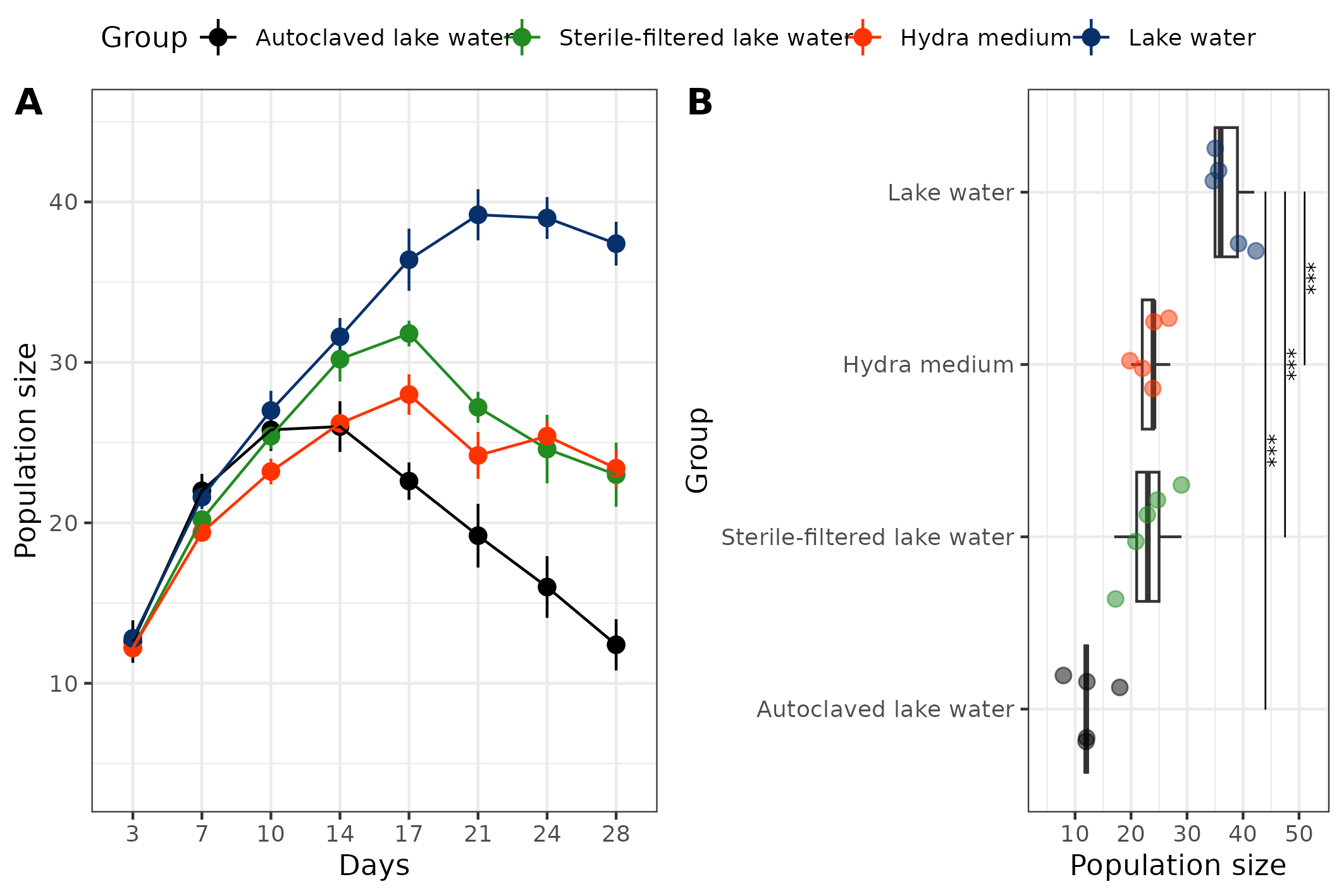


**Supplementary Figure 1.** Population dynamics (A) and final population size (B) of field-collected *Hydra oligactis* in a pilot experiment done in January-February 2022. Polyps were cultured on 8 ºC and a 16/8 hours light/dark cycle either in lake water, autoclaved lake water, sterile-filtered lake water (with a 0.22 μm PES membrane), or a standard hydra medium. All treatment groups consisted of five jars with 10 polyps each as starting population size. Significance stars: *** - p<0.001; ** - p<0.01; * p<0.05; n.s. p≥0.05. Significance was estimated with treatment vs. control post-hoc tests performed after Poisson GLM, with lake water as a reference level.

**
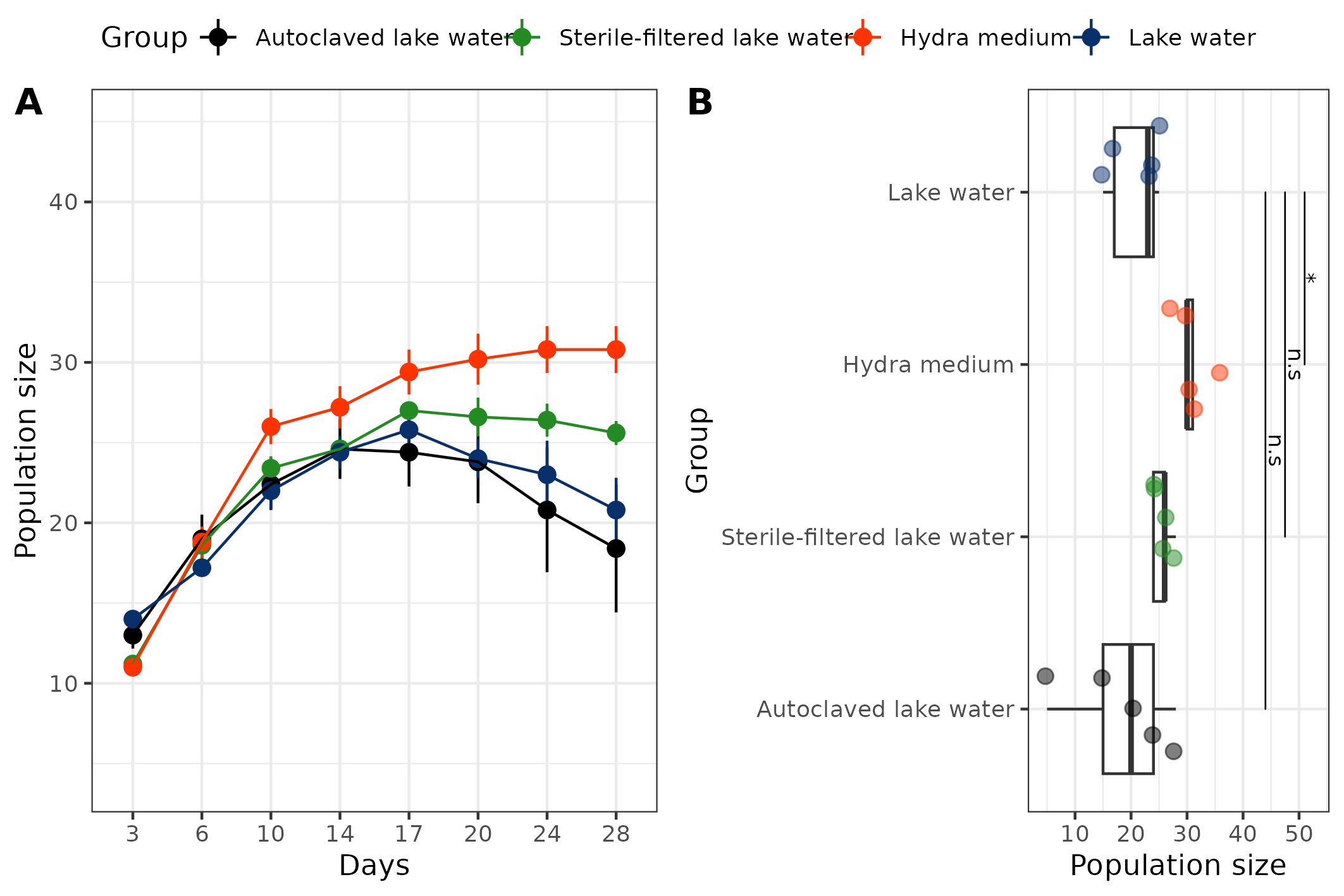
**

**Supplementary Figure 2.** Population dynamics (A) and final population size (B) of field-collected *Hydra oligactis* in a pilot experiment done in March-April 2022. Polyps were cultured on 12 ºC and a 16/8 hours light/dark cycle either in lake water, autoclaved lake water, sterile-filtered lake water (with a 0.22 μm PES membrane), or a standard hydra medium. All treatment groups consisted of five jars with 10 polyps each as starting population size. Significance stars: *** - p<0.001; ** - p<0.01; * p<0.05; n.s. p≥0.05. Significance was estimated with treatment vs. control post-hoc tests performed after Poisson GLM, with lake water as a reference level.


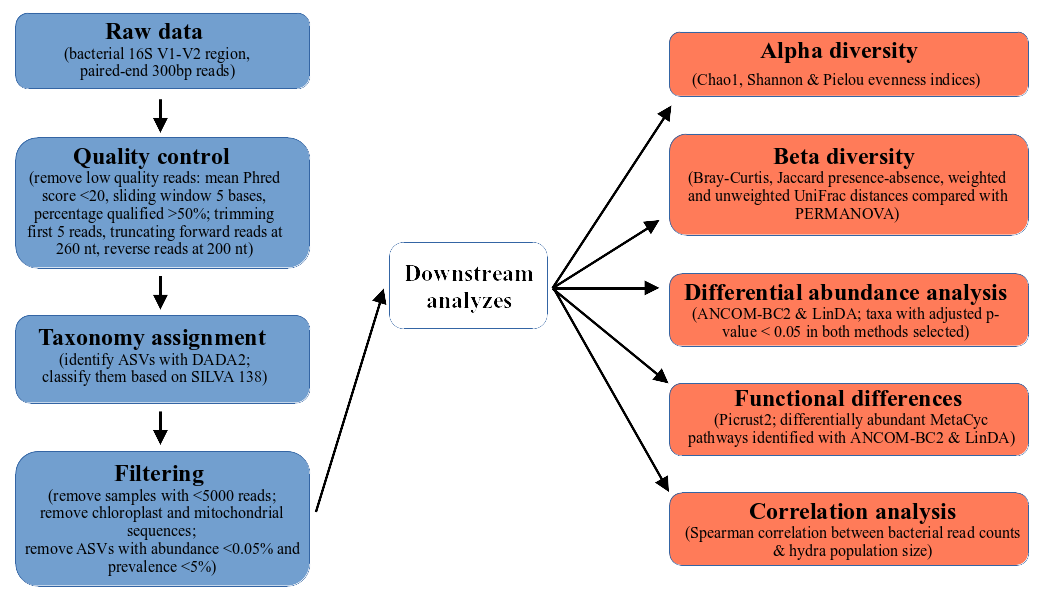
**Supplementary Figure 3.** Schematic representation of the 16S sequencing data analysis pipeline.


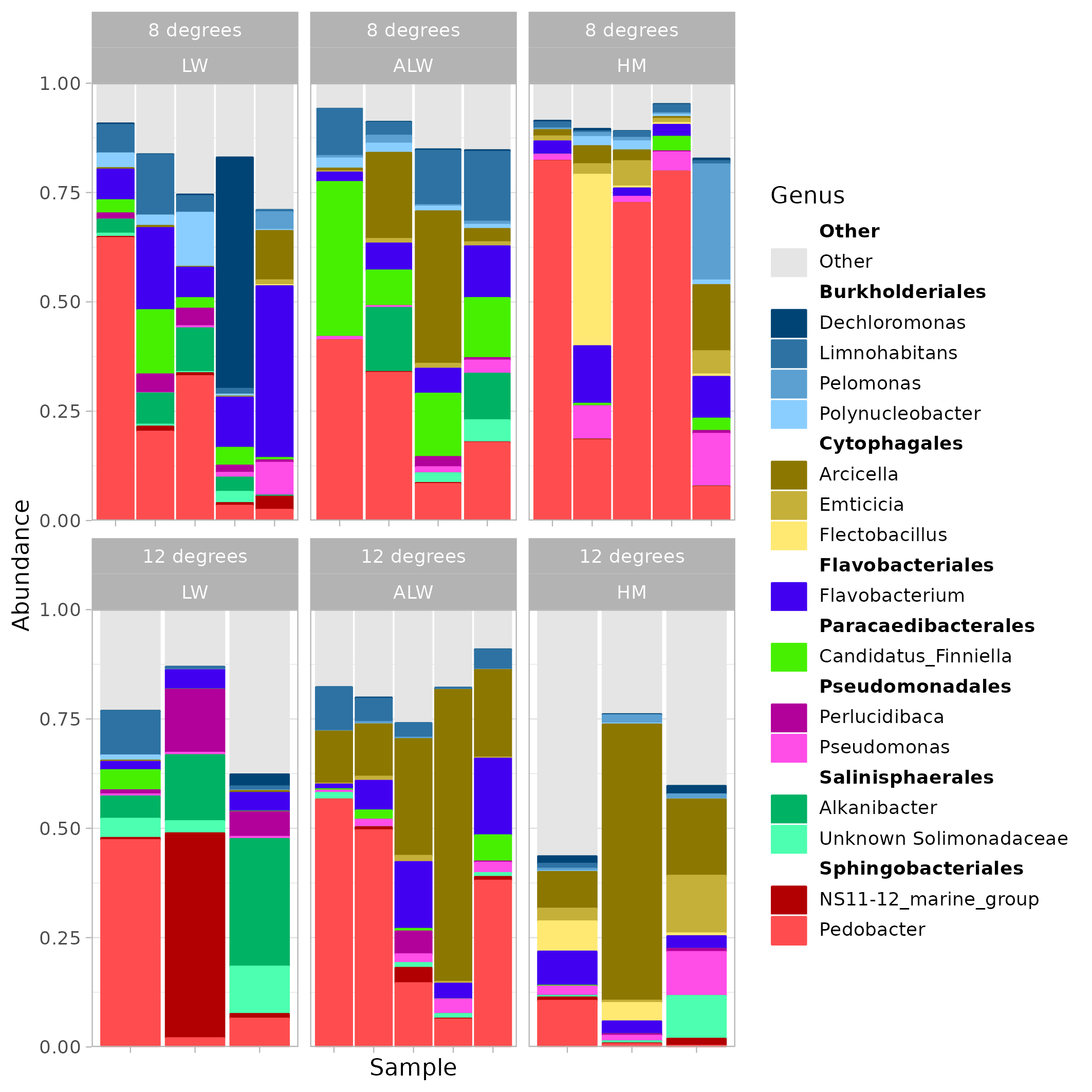


**Supplementary Fig. 4.** ASV abundance of the top 15 most abundant bacterial genera on polyps.


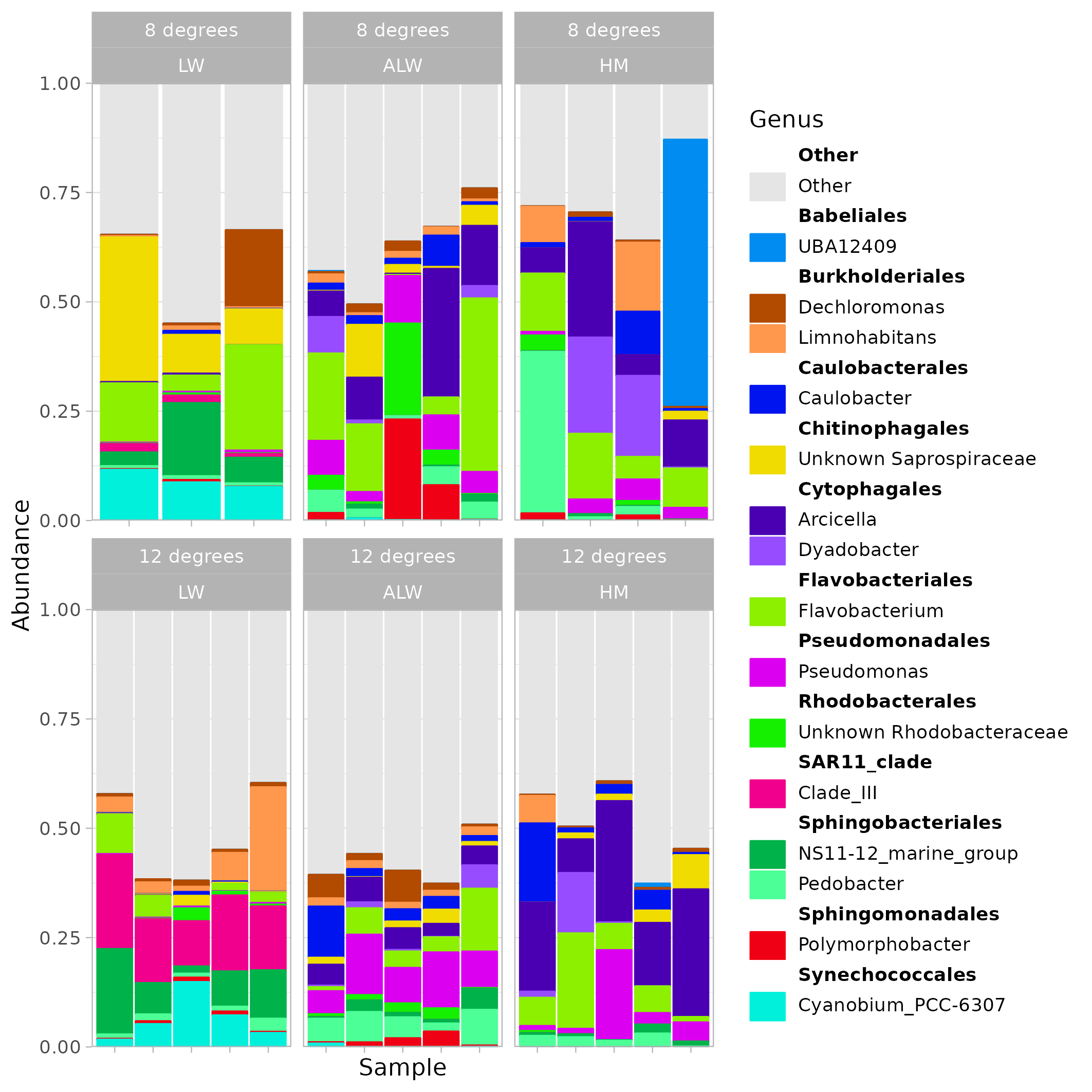
**Supplementary Fig. 5.** ASV abundance of the top 15 most abundant bacterial genera in water.

## Supplementary Tables

**Supplementary Table 1.** Summary of experimental designs

|  | Start date | Temperature | Treatment groups |
| --- | --- | --- | --- |
| Pilot 1 | 10^th^ January 2022 | 8 °C | - Normal lake water,  - Autoclaved lake water  - 0.22 μm filtered lake water  - 0.22 μm filtered Hydra medium |
| Pilot 2 | 18^th^ March 2022 | 12 °C | - Normal lake water,  - Autoclaved lake water  - 0.22 μm filtered lake water  - 0.22 μm filtered Hydra medium |
| Main experiment | 24^th^ January 2023 | 8 and 12 °C | - Normal lake water,  - Autoclaved lake water  - 0.22 μm filtered Hydra medium |

**Supplementary Table 2.** Summary of 16S amplicon sequencing data generated for this study: sample size, Chao1, Shannon and Pielou’s evenness diversity indices for polyp and water samples.

|  |  | Chao1 | | Shannon | | Pielou’s evenness | |
| --- | --- | --- | --- | --- | --- | --- | --- |
| Group | N | Mean | SD | Mean | SD | Mean | SD |
| *Polyp samples* | | | | | | | |
| Field sample | 4 | 65.25 | 13.89 | 2.72 | 0.93 | 0.65 | 0.19 |
| Lake water 8 ºC | 5 | 136.20 | 24.35 | 3.70 | 0.54 | 0.75 | 0.09 |
| Lake water 12 ºC | 3 | 139.33 | 35.23 | 3.50 | 0.83 | 0.71 | 0.13 |
| Autoclaved lake water 8 ºC | 4 | 123.75 | 44.78 | 3.54 | 0.57 | 0.74 | 0.07 |
| Autoclaved lake water 12 ºC | 5 | 122.00 | 27.57 | 3.50 | 0.43 | 0.73 | 0.07 |
| Hydra medium 8 ºC | 5 | 102.00 | 14.73 | 2.92 | 0.59 | 0.63 | 0.12 |
| Hydra medium 12 ºC | 3 | 125.00 | 33.78 | 4.03 | 0.72 | 0.84 | 0.10 |
| *Water samples* |  |  |  |  |  |  |  |
| Field sample | 2 | 112.00 | 29.70 | 4.14 | 0.25 | 0.88 | 0.00 |
| Lake water 8 ºC | 3 | 174.00 | 18.08 | 4.32 | 0.31 | 0.84 | 0.04 |
| Lake water 12 ºC | 5 | 193.20 | 30.57 | 4.63 | 0.27 | 0.88 | 0.03 |
| Autoclaved lake water 8 ºC | 5 | 190.20 | 35.06 | 4.60 | 0.33 | 0.88 | 0.04 |
| Autoclaved lake water 12 ºC | 5 | 248.40 | 24.34 | 5.15 | 0.13 | 0.93 | 0.02 |
| Hydra medium 8 ºC | 4 | 105.50 | 13.18 | 3.68 | 0.54 | 0.79 | 0.11 |
| Hydra medium 12 ºC | 5 | 107.60 | 11.93 | 3.87 | 0.26 | 0.83 | 0.06 |
